# Sequence Variability in Staphylococcal Enterotoxin Genes *seb*, *sec*, and *sed*

**DOI:** 10.3390/toxins8060169

**Published:** 2016-06-01

**Authors:** Sophia Johler, Henna-Maria Sihto, Guerrino Macori, Roger Stephan

**Affiliations:** 1 Institute for Food Safety and Hygiene, Vetsuisse Faculty University of Zurich, Winterthurerstrasse 272, 8057 Zurich, Switzerland; henna-maria.sihto@uzh.ch (H.-M.S.); stephanr@fsafety.uzh.ch (R.S.); 2 National Reference Laboratory for Coagulase-Positive Staphylococci including *Staphylococcus aureus*, Istituto Zooprofilattico Sperimentale del Piemonte, Liguria e Valle d’Aosta, Via Bologna 148, 10154 Torino, Italy; guerrino.macori@izsto.it

**Keywords:** *Staphylococcus aureus*, enterotoxin, sequence variation, *seb*, *sec*, *sed*, promoter

## Abstract

Ingestion of staphylococcal enterotoxins preformed by *Staphylococcus aureus* in food leads to staphylococcal food poisoning, the most prevalent foodborne intoxication worldwide. There are five major staphylococcal enterotoxins: SEA, SEB, SEC, SED, and SEE. While variants of these toxins have been described and were linked to specific hosts or levels or enterotoxin production, data on sequence variation is still limited. In this study, we aim to extend the knowledge on promoter and gene variants of the major enterotoxins SEB, SEC, and SED. To this end, we determined *seb*, *sec,* and *sed* promoter and gene sequences of a well-characterized set of enterotoxigenic *Staphylococcus aureus* strains originating from foodborne outbreaks, human infections, human nasal colonization, rabbits, and cattle. New nucleotide sequence variants were detected for all three enterotoxins and a novel amino acid sequence variant of SED was detected in a strain associated with human nasal colonization. While the *seb* promoter and gene sequences exhibited a high degree of variability, the *sec* and *sed* promoter and gene were more conserved. Interestingly, a truncated variant of *sed* was detected in all tested *sed* harboring rabbit strains. The generated data represents a further step towards improved understanding of strain-specific differences in enterotoxin expression and host-specific variation in enterotoxin sequences.

## 1. Introduction

Staphylococcal food poisoning (SFP) is the most prevalent foodborne intoxication worldwide. The Centers for Disease Control estimate that 240,000 cases per year occur in the US alone, leading to 1000 hospitalizations and six deaths [1]. Upon ingestion, staphylococcal enterotoxins (SEs) secreted by *Staphylococcus (S.) aureus* during growth in the food matrix elicit symptoms of acute gastroenteritis such as violent vomiting and diarrhea [2]. *S. aureus* strains can produce one or several of the five major SEs (SEA, SEB, SEC, SED, SEE). 

Pronounced strain-specific variation of SE mRNA and protein levels has been reported, in particular under conditions of environmental stress encountered in the food matrix [3,4,5,6]. Expression of the phage-encoded SEA was shown to be linked to the life cycle of the phage [7,8] and to nucleotide sequence variation in the *sea* gene and upstream promoter region [9]. 

There is some data on the variation of the enterotoxin gene and promoter sequences of SEB, SEC, and SED. Previous studies characterizing the *seb* and *sed* promoters have shown that the region from −98 to −59 is required for the expression and regulation of *seb* [10] and that the region from −34 to +18 is required for *sed* promoter function [11].

The *seb* gene resides in one of seven different *S. aureus* pathogenicity islands (SaPIs) [12,13,14]. Strains harboring different SaPIs carrying *seb* were reported to vary in SEB levels produced [13]. To date, five different allelic variants of SEB have been described that vary in biological activity [15]. 

The *sec* gene can also be located in different SaPIs, including SaPIn1, SaPIm1, SaPImw2, and SaPIbov1. Four variants of SEC (SEC1-4) associated with human *S. aureus* strains have been described, as well as the host-specific variants SEC-bovine and SEC-ovine [16,17,18,19].

The *sed* gene and reporter sequences seem to be highly conserved and are located on a pIB485-related 27.6 kb plasmid [20]. However, strains harboring a single base deletion in various locations in the *sed* sequence have been reported in *S. aureus* isolates obtained from human hosts [21,22,23,24]. 

The aim of this study was to analyze promoter and gene sequences of *seb*, *sec*, and *sed* from *S. aureus* strains originating from different sources. Data on the variability of enterotoxin nucleotide sequences in strains from different hosts can represent an important further step in understanding strain-specific variation in SE expression, and in monitoring the evolution of *S. aureus* pathogenicity and host adaptation.

## 2. Results

### 2.1. Seb Promoter and Gene Sequences

The *seb* promoter and gene sequences of 12 strains were determined and alignments of all sequences are provided as a supplementary file (Figure S1). Five variants of the *seb* promoter (*seb*_p_ v1–v5) were detected that differed at several nucleotide positions. While −35 (TGAATA) and −10 (TATATT) *seb* promoter elements were identical in all tested strains, sequence variation was detected in the region essential for *seb* expression that is located between 59 and 93 nucleotides upstream of the transcription start site. The *seb* promoter variants *seb*_p_ v1, v2, and v5 exhibited nucleotides GT (positions −47, −46), AT (positions −23, −22), and A (position −18), while *seb*_p_ v3 and v4 exhibited nucleotides AA (position −47, −46), GA (positions −23, −22), and G (position −18). Promoter variant *seb*_p_ v4 and v5 did not correspond to any known *seb* promoter sequences in GenBank.

**Figure 1:**
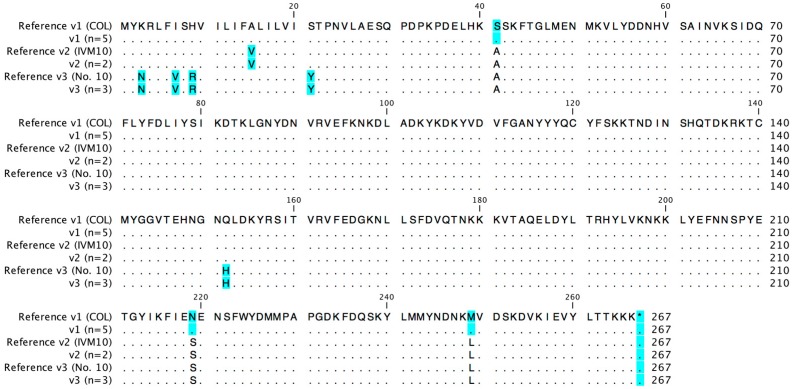
Amino acid variants of SEB. Amino acid exchanges compared to the most common amino acid detected are highlighted in blue (*n* = number of strains representing each variant).

The *seb* gene ORF exhibited a length of 801 bp in all 12 strains. Nucleotide sequence variation was found at numerous positions (9, 19, 26, 44, 52, 62, 84, 87, 121, 162, 165, 351, 393, 405, 456, 484, 513, 522, 543, 621, 656, 738, 745), leading to the identification of four different variants (*seb* v1–v4). Two strains (RKI4 and SAI33) harbored the novel variant v3. The different *seb* nucleotide sequences resulted in three different amino acid variants (266 amino acid precursor), which were identical to known amino acid variants of reference strains (COL, IVM10, No. 10). An alignment of the respective amino acid sequences and reference sequences is provided in Figure 1.

Screening of strains representing the different *seb* variants for production of SEB using SET-RPLA (Oxoid, Pratteln, Switzerland) showed that all variants are expressed.

### 2.2. Sec Promoter and Gene Sequences

The *sec* promoter and gene sequences were determined in 10 strains and alignments of all sequences are provided as a supplementary file (Figure S2). The −35 (TTGAA) and −10 (TATATTT) *sec* promoter elements were identical in all tested strains. 

**Figure 2:**
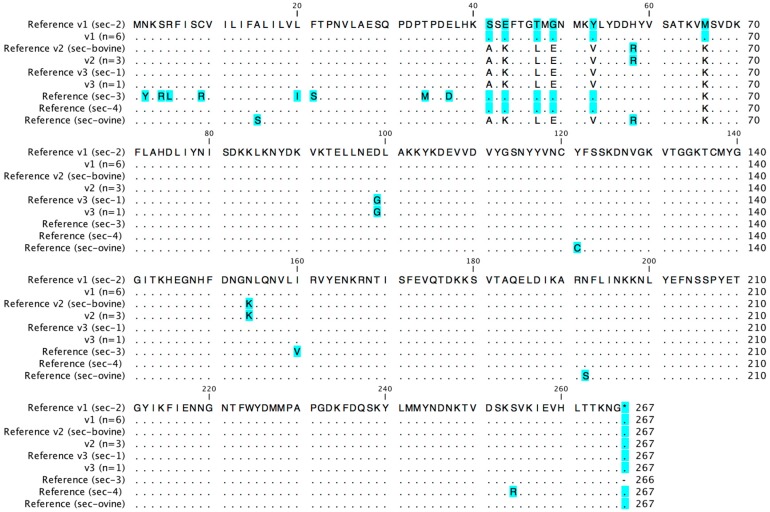
Amino acid variants of SEC. Amino acid exchanges compared to the most common amino acid detected are highlighted in blue (*n* = number of strains representing each variant).

The *sec* ORF exhibited a length of 801 bp in all strains. Isolates obtained from nasal colonization and foodborne outbreaks harbored a *sec* variant (v1) identical to the previously described SEC-2 subtype. All bovine strains exhibited *sec* v2 identical with the SEC-bovine subtype. For SAI3, a human infection isolate, a *sec* variant (*sec* v3) identical to subtype SEC-1 was found. For SAI48, a strain also linked to an infection in a human patient, a novel nucleotide sequence similar to SEC-2, with the exception of a point mutation at position 87 (T -> C), was identified. The four nucleotide sequence variants resulted in three different predicted variants of the 266-amino-acid precursor. Nucleotide sequence variants v1 and the novel variant v4 both resulted in the amino acid variant *sec*_aa_ v1 (SEC-2), while v2 resulted in *sec*_aa_ v2 (SEC-bovine), and v3 resulted in *sec*_aa_ v3 (SEC-1), respectively. An alignment of the respective amino acid sequences and reference sequences is provided as Figure 2.

Screening the strains exhibiting *sec* for SEC production using SET-RPLA led to detection of SEC in all strains, showing that all *sec* variants are expressed. 

### 2.3. Sed Promoter and Gene Sequences

The sequences of the *sed* promoter and gene were determined in 12 strains and alignments of all sequences are provided as a supplementary file (Figure S3). The −35 (ATGAAA) and −10 (TATAA) promoter elements were identical in all tested strains. 

**Figure 3:**
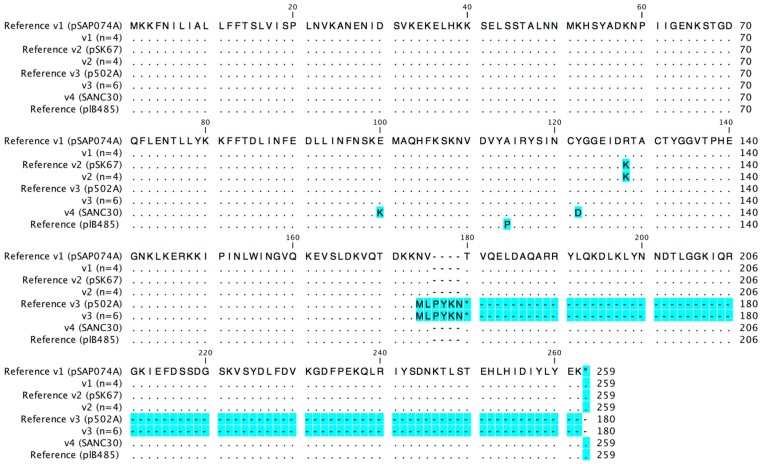
Amino acid variants of SED. Amino acid exchanges compared to the most common amino acid detected are highlighted in blue (*n* = number of strains representing each variant).

The *sed* sequences were also highly conserved. However, point mutations were observed in strain SANC30 (position 198 G -> A, position 364 T -> G) and in strains BW10, RKI1, RKI2, and SAR35 (position 383 G -> A). In total, four different amino acid variants were detected, none of which was 100% identical to the common *sed* plB485 reference sequence (Genbank accession number M28521.1). SANC30 exhibited a novel amino acid sequence (variant 4) with two amino acid changes (position 100 E -> K, position 121 Y -> D) that did not correspond to any sequence in the GenBank database. An alignment of the respective amino acid sequences and reference sequences is provided in Figure 3.

All three tested rabbit isolates harbored the same *sed* nucleotide sequence variant (v3), which exhibits a deletion in *sed* at nucleotide position 521, resulting in a premature stop codon at amino acid position 180. To confirm the possibility of a host-specific variant, an additional three *sed+* rabbit strains were sequenced, which harbored the same truncated variant.

Screening of strains representing the different *sed* variants for production of SED by SET-RPLA showed that not only the complete *sed* variants but also the truncated *sed* v3 were expressed. However, for the strains harboring the truncated *sed* variant, SED levels were far lower, with only the first dilution in the dilution series yielding a weakly positive test result. Four rabbit strains harboring the truncated *sed* v3 variant were screened. While SED was detected in three of these strains (SAK8, SAK11, SAK13), one strain (SAK64) did not yield a positive result for SED production. While all *sed+* rabbit strains represent the same clonal complex (CC5), SAK64 was the only rabbit strain of *spa* type t160.

## 3. Discussion

In this study, several new variants of *seb, sec,* and *sed* enterotoxin genes and promoters were detected. Sequencing of *seb,* *sec*, and *sed* promoter regions revealed that promoter sequences were highly conserved in *sec* and *sed*. In contrast, several variable positions were observed in the *seb* promoter region, including the region required for *seb* transcription and expression. This is consistent with findings by Sato’o *et al.* [13], reporting high variability in *seb* upstream sequences from different strains. In the same study, several novel SaPIs carrying *seb* were identified and linked to differences in SEB production levels. However, the differences in *seb* promoter regions did not correlate with SEB production in a statistically significant manner [13]. 

Comparative analysis of the nucleotide sequences of the *seb*, *sec*, and *sed* genes showed that *sed* sequences were more conserved than *seb* and *sec* sequences. The length of the *seb* and *sec* coding sequences determined in this study was consistent with previous reports [18,25,26]. 

Most of the residues that are conserved throughout all SEs are either centrally located or can be found at the *C*-terminal end [27]. This is also consistent with the findings in this study for the amino acid prediction of *seb,* and *sec* variants, indicating that amino acid exchanges were more likely to occur at the *N*-terminus. However, for SED*,* the highest degree of amino acid variability detected in this study was centrally located*.*

Predicting altered functionality based on the detected amino acid exchanges is challenging, as emetic activity is still poorly understood. While lack of the disulfide loop was suggested to result in no or lower emetic activity, it has been shown that the disulfide bond is not a prerequisite for emetic activity [28]. Concerning superantigenic activity, ovine and bovine SEC variants were reported to be strongly altered in function due to only three amino acid changes resulting in host-dependent superantigenicity [27]. With regard to antigenicity, it was shown that SE variants differing in several residues, such as the SEC variants identified in FRI909 and FRI913 (9 differing residues), can still be antigenetically indistinguishable [27].

For SEC, four variants (SEC1-4) associated with human *S. aureus* isolates have been reported, as well as the host-specific variants SEC-bovine and SEC-ovine [16,17,18,19]. In contrast, subtypes of *seb* and the different SaPIs associated with these subtypes have only recently gained attention [13,15]. Kohler *et al.* demonstrated the existence of multiple SEB variants that differed in their ability to activate subsets of T cells and in their effects on the proliferation of peripheral blood mononuclear cells and rabbit splenocytes [15].

In this study, a novel *seb* nucleotide sequence variant (*seb* v3) was identified in two of the tested strains (RKI4 and SAI33). However, the *seb* v3 nucleotide sequence variant results in a known SEB amino acid sequence identical to reference strain No. 10. For *sec*, one novel *sec* nucleotide sequence (*sec* v4) was detected in one strain (SAI48). Amino acid sequence prediction showed that *sec* v4 results in an amino acid sequence identical to the one of *sec* v1, which is also known as SEC-2 (reference strain 79_S10). For *sed*, one novel nucleotide sequence (*sed* v4) was determined in a strain associated with human nasal colonization (SANC30). The novel *sed* v4 nucleotide sequence variant results in a novel amino acid sequence variant of SED that was not previously described elsewhere (*sed_aa_* v4). 

In this study, a variant of *sed* was identified which was present in all tested rabbit isolates (*n* = 6). This variant *sed* v3 exhibited a deletion that resulted in a premature stop codon and a truncated *sed* amino acid precursor. In foodborne outbreak isolates, deletions at nucleotide positions 150 [21] and 514 [22] resulting in a premature stop codon have been reported. A deletion in *sed* identical to the one seen in the rabbit isolates in this study (nucleotide position 521) has been reported in *S. aureus* isolates originating from humans and from food [23,24]. While Lis *et al.* confirmed transcription of *sed* by qPCR, they could not detect SED protein by ELISA or Western blotting [24]. In contrast, in this study, three of four rabbit strains tested with truncated *sed* variants yielded a weak, but positive result for SED in the SET-RPLA assay. The deletion in *sed* may impair the functionality of the protein and recognition by various detection methods.

## 4. Conclusions

The sequence data generated in this study extends the current knowledge on sequence variation in enterotoxin genes of *S. aureus* strains isolated from various sources. Several novel variants of enterotoxin promoter and gene nucleotide sequences were described, and a novel amino acid sequence variant of SED was identified in a strain obtained from a nasal carrier. In addition, the results presented in this study confirm previous reports of host-specific enterotoxin variants such as SEC-bovine. Interestingly, all *sed+* rabbit strains tested in this study harbored a *sed* variant that exhibited a deletion in *sed* leading to a premature stop codon. 

The data generated represents a further step towards improved understanding of strain-specific differences in enterotoxin expression and host-specific variation in enterotoxin sequences.

## 5. Materials and Methods 

### 5.1. Bacterial Strains 

The *S. aureus* isolates used in this study originated from SFP outbreaks, asymptomatic nasal colonization or cases of infections in humans, as well as rabbit carcasses and bovine mastitis milk. Isolates were selected from a large collection of well-characterized *S. aureus* strains*,* for which DNA microarray enterotoxin hybridization patterns, *spa* types, and clonal complexes had been previously determined and published [14,28,29,30,31]. Detailed information on all *S. aureus* strains used in this study is provided in Table 1.

### 5.2. DNA Extraction and PCR Amplification 

Frozen stock cultures (−80 °C) of *S. aureus* strains were resuscitated by plating on 5% sheep blood agar and incubation at 37 °C over night. Bacterial DNA was extracted using the DNeasy Blood and Tissue Kit (Qiagen, Hilden, Germany) following the manufacturer′s instructions.

PCR was performed using the Phusion High-Fidelity System (Thermo Scientific, Reinach, Switzerland) using a total reaction volume of 50 μL. All primers and primer-pair specific annealing temperatures are provided as supplemental material (Table S1). For each reaction, 5 μL buffer, 2 μL DMSO, 1 μL dNTP mix, 2 μL of each primer (*c* = 10 μM), 0.5 μL Phusion High-Fidelity DNA polymerase, 36.5 μL Aq.B., and 1 μL DNA template were used. PCR cycling conditions included: 5 min hot start at 95 °C, followed by 30 amplification cycles (denaturation at 95 °C for 30 s, annealing at the primer-specific annealing temperature for 30 s, elongation at 72 °C for 75 s), a final elongation step at 72 °C for 10 min, and a cooling step. Target-specific amplification was confirmed by electrophoresis using a 1% agarose gel. 

### 5.3. PCR Purification and Sequencing

PCR amplicons were purified using the MinElute PCR Purification Kit (Qiagen, Hilden, Germany) and sequencing was outsourced (Microsynth, Balgach, Switzerland). The acquired sequences were analyzed using CLC Main Workbench software (Version 6.9, CLC Bio/Qiagen, Aarhus, Denmark, 2012) and were compared to reference nucleotide sequences imported from GenBank (NCBI). Novel variants of enterotoxin promoter or gene sequences were subsequently submitted to GenBank. 

### 5.4. Toxin Detection by SET-RPLA

Expression of different *seb*, *sec*, and *sed* variants was assessed in selected strains using the SET-RPLA kit (Oxoid). Enterotoxins were detected using bacterial culture filtrates (0.2 μm filter, Whatman, Sigma-Aldrich, Buchs, Switzerland) from stationary phase cultures of each strain in Luria Bertrani (LB, Becton Dickinson, Allschwil, Switzerland) broth (37 °C, 225 rpm shaking, 20 h of incubation) in accordance with the manufacturer’s instructions. Culture filtrates were diluted in a five-fold dilution series for semi-quantitative detection of SEB, SEC, and SED. For *seb,* strains KLT6, SANC49, SANC14, SAI45, RKI4, and SAI40 were tested for SEB expression. All *sec* strains were assayed for SEC expression. For *sed,* strains BW10, RKI1, RKI2, KLT8, SAK8, SAK11, SAK13, and SAK64 were tested for SED expression.

### 5.5. Amino Acid Identity

A pairwise amino acid identity comparison between all SEB, SEC, and SED enterotoxin variants is provided in Table 2. 

### 5.6. Accession Numbers

All variants of promoter and gene sequences were submitted to GenBank and can be accessed using accession numbers KX168612–KX168635. Promoter sequence variants are available for *seb* variants (*seb*_p_ v1 = KX168623, *seb*_p_ v2 = KX168624, *seb*_p_ v3 = KX168625, *seb*_p_ v4 = KX168626, *seb*_p_ v5 = KX168627), *sec* variants (*sec*_p_ v1 = KX168633, *sec*_p_ v2 = KX168634, *sec*_p_ v3 = KX168635), and *sed* variants (*sed*_p_ v1 = KX168616, *sed*_p_ v2 = KX168617, *sed*_p_ v3 = KX168618). Sequence variants are available for the *seb* gene (*seb* v1 = KX168628, *seb* v2 = KX168629, *seb* v3 = KX168630, *seb* v4 = KX168631, *seb* v5 = KX168632), the *sec* gene (*sec* v1 = KX168612, *sec* v2 = KX168613, *sec* v3 = KX168614, *sec* v4 = KX168615), and the *sed* gene (*sed* v1 = KX168619, *sed* v2 = KX168620, *sed* v3 = KX168621, *sed* v4 = KX168622).

## Figures and Tables

**Table 1:** Detailed overview of sequence variants of enterotoxin promoters and genes of all *S. aureus* strains used in this study. In addition, information on other major enterotoxin genes harbored by each strain, the source of the strain, and its assignment to a *spa* type and clonal complex are provided.

Gene	Strain ID	Identical Reference ^1^	Promoter Variant ^2^ (Reference)	Gene Variant ^2^ (Reference)	Amino Acid Variant (Reference)	Source	Clonal Complex/*spa* Type	Reference
*seb*	KLT6	COL	*seb*_p_ v1	*seb* v1	*seb*_aa_ v1	SFP ^3^	CC12/t160	[29]
	SANC31	COL	*seb*_p_ v1	*seb* v1	*seb*_aa_ v1	Human nasal colonization	CC59/t216	[30]
	SANC49	COL	*seb*_p_ v1	*seb* v1	*seb*_aa_ v1	Human nasal colonization	CC59/t216	[30]
	SAK9	COL	*seb*_p_ v1	*seb* v1	*seb*_aa_ v1	Rabbit	CC5/t8456	[31]
	SAK18	COL	*seb*_p_ v1	*seb* v1	*seb*_aa_ v1	Rabbit	CC5/t8456	[31]
	SAI10	COL	*seb*_p_ v1	*seb* v1	*seb*_aa_ v1	Human infection	CC59/t216	[30]
	SAI50	COL	*seb*_p_ v1	*seb* v1	*seb*_aa_ v1	Human infection	CC59/t015	[30]
	SANC14	IVM10	*seb*_p_ v2	*seb* v2	*seb*_aa_ v2	Human nasal colonization	CC45/t630	[30]
	RKI4	novel ^4^	*seb*_p_ v3 (No. 10)	*seb* v3 (novel) ^4^	*seb*_aa_ v3 (No. 10)	SFP	CC9/t733	[14]
	SAI40	novel ^4^	*seb*_p_ v4 (novel) ^4^	*seb* v3 (No. 10)	*seb*_aa_ v3 (No. 10)	Human infection	CC15/t084	[30]
	SAI33	novel ^4^	*seb*_p_ v4 (novel) ^4^	*seb* v3 (novel) ^4^	*seb*_aa_ v3 (No. 10)	Human infection	CC20/t164	[30]
	SAI45	novel ^4^	*seb*_p_ v5 (novel) ^4^	*seb* v4 (IVM10)	*seb*_aa_ v2 (IVM10)	Human infection	CC121/t272	[30]
*sec*	BW10	79_S10	*sec*_p_ v1	*sec* v1 = SEC-2	*sec*_aa_ v1 = SEC-2	SFP	CC45/t383	Medical Department of the German Federal Armed Forces, Germany
	LRA1	79_S10	*sec*_p_ v1	*sec* v1 = SEC-2	*sec*_aa_ v1 = SEC-2	SFP	CC73/t015	Bavarian State Office of Health and Food Safety, Germany
	SANC23	79_S10	*sec*_p_ v1	*sec* v1 = SEC-2	*sec*_aa_ v1 = SEC-2	Human nasal colonization	CC8/t8016	[30]
	SANC48	79_S10	*sec*_p_ v1	*sec* v1 = SEC-2	*sec*_aa_ v1 = SEC-2	Human nasal colonization	CC45/t015	[30]
	NB6	79_S10	*sec*_p_ v1	*sec* v1 = SEC-2	*sec*_aa_ v1 = SEC-2	SFP	CC45/t6969	Bavarian State Office of Health and Food Safety, Germany
	SAR1	RF122	*sec*_p_ v2	*sec* v2 = SEC-bovine	*sec*_aa_ v2 = SEC-bovine	Bovine mastitis milk	CC151/t529	[32]
	SAR38	RF122	*sec*_p_ v2	*sec* v2 = SEC-bovine	*sec*_aa_ v2 = SEC-bovine	Bovine mastitis milk	CC151/t529	[32]
	SAR50	RF122	*sec*_p_ v2	*sec* v2 = SEC-bovine	*sec*_aa_ v2 = SEC-bovine	Bovine mastitis milk	CC151/t529	[32]
	SAI3	novel ^4^	*sec*_p_ v3 (H-EMRSA-15)	*sec* v3 = SEC-1 (B1085)	*sec*_aa_ v3 = SEC-1 (B1085)	Human infection	CC8/t148	[30]
	SAI48	novel ^4^	*sec*_p_ v1 (79_S10)	*sec* v4 (novel) ^4^	*sec*_aa_ v1 = SEC-2 (79_S10)	Human infection	CC5/t002	[30]
*sed*	KLT8	pSAP074A	*sed*_p_ v1	*sed* v1	*sed*_aa_ v1	SFP	CC5/t8017	Cantonal Laboratory Thurgau, Switzerland
	SAI8	pSAP074A	*sed*_p_ v1	*sed* v1	*sed*_aa_ v1	Human infection	CC5/t954	[30]
	SAI41	pSAP074A	*sed*_p_ v1	*sed* v1	*sed*_aa_ v1	Human infection	CC5/t8017	[30]
	SAI48	novel ^4^	*sed*_p_ v3 (novel) ^4^	*sed* v1 (pSAP074A)	*sed*_aa_ v1 (pSAP074A)	Human infection	CC5/t002	[30]
	BW10	pSK67	*sed*_p_ v2	*sed* v2	*sed*_aa_ v2	SFP	CC45/t383	Medical Department of the German Federal Armed Forces, Germany
	RKI1	pSK67	*sed*_p_ v2	*sed* v2	*sed*_aa_ v2	SFP	CC8/t648	Robert Koch Institute, Germany
	RKI2	pSK67	*sed*_p_ v2	*sed* v2	*sed*_aa_ v2	SFP	CC8/t008	Robert Koch Institute, Germany
	SAR35	novel ^4^	*sed*_p_ v1 (pSAP074A)	*sed* v2 (pSK67)	*sed*_aa_ v2 (pSK67)	Bovine mastitis milk	CC8/t2953	[32]
	SAK8	novel ^4^	*sed*_p_ v1 (pSAP074A)	*sed* v3 (p502A)	*sed*_aa_ v3 (p502A)	Rabbit	CC5/t179	[31]
	SAK9	novel ^4^	ND ^5^	*sed* v3 (p502A)	*sed*_aa_ v3 (p502A)	Rabbit	CC5/t8456	[31]
	SAK11	novel ^4^	*sed*_p_ v1 (pSAP074A)	*sed* v3 (p502A)	*sed*_aa_ v3 (p502A)	Rabbit	CC5/t179	[31]
	SAK13	novel ^4^	*sed*_p_ v1 (pSAP074A)	*sed* v3 (p502A)	*sed*_aa_ v3 (p502A)	Rabbit	CC5/t179	[31]
	SAK18	novel ^4^	ND ^5^	*sed* v3 (p502A)	*sed*_aa_ v3 (p502A)	Rabbit	CC5/t8456	[31]
	SAK64	novel ^4^	ND ^5^	*sed* v3 (p502A)	*sed*_aa_ v3 (p502A)	Rabbit	CC5/t160	[31]
	SANC30	novel ^4^	*sed*_p_ v1 (pSAP074A)	*sed* v4 (novel)^4^	*sed*_aa_ v4 (novel) ^4^	Human nasal colonization	CC5/t002	[30]

^1^ Reference sequences were obtained from GenBank. Accession numbers: CP000046.1 (strain COL), AB716349.1 (strain IVM10), AB716351.1 (strain No. 10), CP010952.1 (strain 93b_S9), CP010944.1 (strain 79_S10), AJ938182.1 (strain RF122), KF386012.1 (strain B1085), CP007659.1 (strain H-EMRSA-15), GQ900426.1, (plasmid pSAP074A), CP007455.1 (plasmid p502A), GQ900447.1 (plasmid pSK67); ^2^ Nucleotide sequence; ^3^ SFP = Staphylococcal Food Poisoning; ^4^ Novel variant with no identical reference sequence available in GenBank; ^5^ Not determined.

**Table 2:** Overview of pairwise amino acid identity of the different SEB, SEC, and SED variants. The novel variant 4 of SED (*sed*_aa_ v4) was detected in a strain isolated from a nasal carrier and has not been previously described.

Variant	SEB v1	SEB v2	SEB v3	SEC-1	SEC-2	SEC-3	SEC-4	SEC-bovine	SEC-ovine	SED v1	SED v2	SED v3	SED v4
SEB v1	100	99	97	68	67	69	67	69	67	36	35	25	36
SEB v2	-	100	98	67	66	67	66	68	67	36	36	25	36
SEB v3	-	-	100	67	66	67	66	68	67	36	35	24	35
SEC-1	-	-	-	100	97	94	97	99	98	32	32	22	32
SEC-2	-	-	-	-	100	96	99	97	96	33	32	22	32
SEC-3	-	-	-	-	-	100	96	94	93	33	33	23	33
SEC-4	-	-	-	-	-	-	100	97	96	33	33	22	33
SEC-bovine	-	-	-	-	-	-	-	100	99	32	32	22	32
SEC-ovine	-	-	-	-	-	-	-	-	100	32	32	21	32
SED v1	-	-	-	-	-	-	-	-	-	100	99	66	99
SED v2	-	-	-	-	-	-	-	-	-	-	100	66	99
SED v3	-	-	-	-	-	-	-	-	-	-	-	100	65
SED v4	-	-	-	-	-	-	-	-	-	-	-	-	100

## Supplementary Materials

The following are available online at www.mdpi.com/2072-6651/8/6/169/s1, Figure S1: Sequence alignments for *seb*. Figure S2: Sequence alignments for *sec*. Figure S3: Sequence alignments for *sed*. Table S1: Primers used in this study.
