# Supplementary material for: Sequence Variability in Staphylococcal Enterotoxin Genes seb, sec, and sed
Source: Toxins (Basel). 2016 Jun 1;8(6):169. doi: 10.3390/toxins8060169 (PMC4926136; doi:10.3390/toxins8060169)
Supplement: Supplementary file 1 [file toxins-08-00169-s001.pdf]

Supplementary Materials: Sequence Variability in Staphylococcal Enterotoxin Genes *seb*, *sec*, and *sed*

Sophia Johler, Henna-Maria Sihto, Guerrino Macori and Roger Stephan

SEB PROMOTER

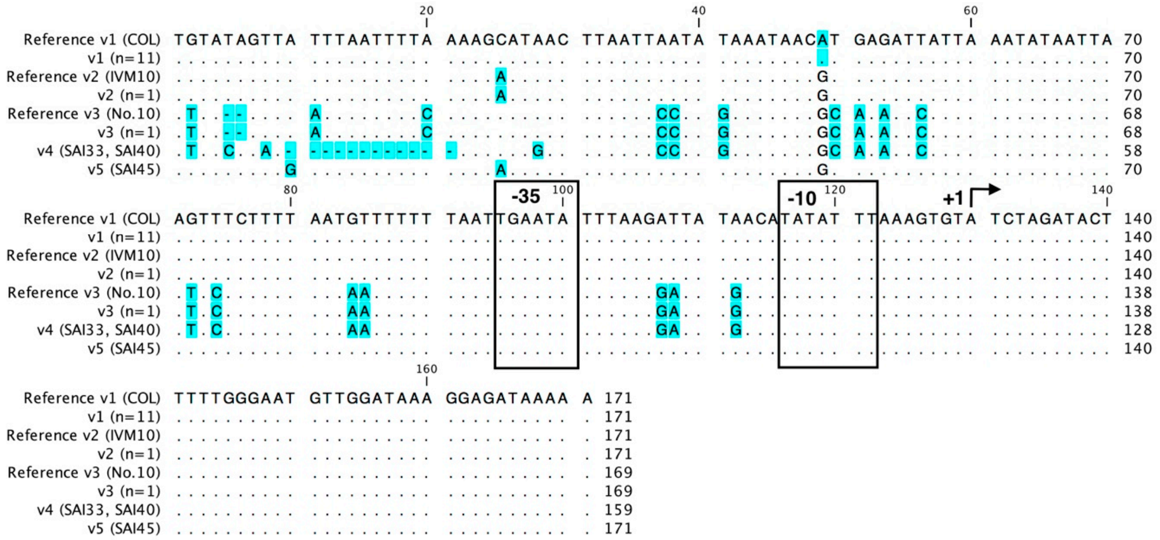

SEB GENE

```
                                         20                    40                    60
Reference v1 (COL)   ATGTATAAGA GATTATTTAT TTCACATGTA ATTTTGATAT TCGCACTGAT ATTAGTTATT TCTACACCCA 70
v1 (n=5)             .......... .......... .......... .......... .......... .......... .......... 70
Reference v2 (IVM10) .......... .......... .......... .......... ...T...... .......... .......... 70
v2 (n=1)             .......... .......... .......... .......... ...T...... .......... .......... 70
Reference v3 (No. 10) ........T. ........G. .....G.... .......... .......... .C........ .A........ 70
v3 (n=1)             ........T. ........G. .....G.... .......... .......... .C........ .A........ 70
Reference v4 (93b_S9) .......... .......... .......... .......... ...T...... .......... .......... 70
v4 (n=1)             .......... .......... .......... .......... ...T...... .......... .......... 70
v5 (RKI4, SAI33)     ........T. ........G. .....G.... .......... .......... .C........ .A........ 70

                               80                   100                   120                   140
Reference v1 (COL)   ACGTTTTAGC AGAGAGTCAA CCAGATCCTA AACCAGATGA GTTGCACAAA TCGAGTAAAT TCACTGGTTT 140
v1 (n=5)             .......... .......... .......... .......... .......... .......... .......... 140
Reference v2 (IVM10) .......... .......... .......... .......... .......... G......... .......... 140
v2 (n=1)             .......... .......... .......... .......... .......... G......... .......... 140
Reference v3 (No. 10) .......... ...A..C... .......... .......... .......... G......... .......... 140
v3 (n=1)             .......... ...A..C... .......... .......... .......... G......... .......... 140
Reference v4 (93b_S9) .......... .......... .......... .......... .......... G......... .......... 140
v4 (n=1)             .......... .......... .......... .......... .......... G......... .......... 140
v5 (RKI4, SAI33)     .......... ...A..C... .......... .......... .......... G......... .......... 140

                                        160                   180                   200
Reference v1 (COL)   GATGGAAAAT ATGAAAGTTT TGTATGATGA TAATCATGTA TCAGCAATAA ACGTTAAATC TATAGATCAA 210
v1 (n=5)             .......... .......... .......... .......... .......... .......... .......... 210
Reference v2 (IVM10) .......... .......... .......... .......... .......... .......... .......... 210
v2 (n=1)             .......... .......... .......... .......... .......... .......... .......... 210
Reference v3 (No. 10) .......... .......... .A..C..... .......... .......... .......... .......... 210
v3 (n=1)             .......... .......... .A..C..... .......... .......... .......... .......... 210
Reference v4 (93b_S9) .......... .......... .......... .......... .......... .......... .......... 210
v4 (n=1)             .......... .......... .......... .......... .......... .......... .......... 210
v5 (RKI4, SAI33)     .......... .......... .A..C..... .......... .......... .......... .......... 210

                               220                   240                   260                   280
Reference v1 (COL)   TTTCTATACT TTGACTTAAT ATATTCTATT AAGGACACTA AGTTAGGGAA TTATGATAAT GTTCGAGTCG 280
v1 (n=5)             .......... .......... .......... .......... .......... .......... .......... 280
Reference v2 (IVM10) .......... .......... .......... .......... .......... .......... .......... 280
v2 (n=1)             .......... .......... .......... .......... .......... .......... .......... 280
Reference v3 (No. 10) .......... .......... .......... .......... .......... .......... .......... 280
v3 (n=1)             .......... .......... .......... .......... .......... .......... .......... 280
Reference v4 (93b_S9) .......... .......... .......... .......... .......... .......... .......... 280
v4 (n=1)             .......... .......... .......... .......... .......... .......... .......... 280
v5 (RKI4, SAI33)     .......... .......... .......... .......... .......... .......... .......... 280

                                        300                   320                   340
Reference v1 (COL)   AATTTAAAAA CAAAGATTTA GCTGATAAAT ACAAAGATAA ATACGTAGAT GTGTTTGGAG CTAATTATTA 350
v1 (n=5)             .......... .......... .......... .......... .......... .......... .......... 350
Reference v2 (IVM10) .......... .......... .......... .......... .......... .......... .......... 350
v2 (n=1)             .......... .......... .......... .......... .......... .......... .......... 350
Reference v3 (No. 10) .......... .......... .......... .......... .......... .......... .......... 350
v3 (n=1)             .......... .......... .......... .......... .......... .......... .......... 350
Reference v4 (93b_S9) .......... .......... .......... .......... .......... .......... .......... 350
v4 (n=1)             .......... .......... .......... .......... .......... .......... .......... 350
v5 (RKI4, SAI33)     .......... .......... .......... .......... .......... .......... .......... 350

                               360                   380                   400                   420
Reference v1 (COL)   TTATCAATGT TATTTTTCTA AAAAAACGAA TGATATTAAT TCGCATCAAA CTGACAAACG AAAAACTTGT 420
v1 (n=5)             .......... .......... .......... .......... .......... .......... .......... 420
Reference v2 (IVM10) C......... .......... .......... .......... ..A....... .......... .......... 420
v2 (n=1)             C......... .......... .......... .......... ..A....... .......... .......... 420
Reference v3 (No. 10) .......... .......... .......... .......... ..A....... ....T..... .......... 420
v3 (n=1)             .......... .......... .......... .......... ..A....... ....T..... .......... 420
Reference v4 (93b_S9) C......... .......... .......... .......... ..A....... .......... .......... 420
v4 (n=1)             C......... .......... .......... .......... ..A....... .......... .......... 420
v5 (RKI4, SAI33)     .......... .......... .......... .......... ..A....... .......... .......... 420

                                        440                   460                   480
Reference v1 (COL)   ATGTATGGTG GTGTAACTGA GCATAATGGA AACCAATTAG ATAAATATAG AAGTATTACT GTTCGGGTAT 490
v1 (n=5)             .......... .......... .......... .......... .......... .......... .......... 490
Reference v2 (IVM10) .......... .......... .......... .......... .......... .......... .......... 490
v2 (n=1)             .......... .......... .......... .......... .......... .......... .......... 490
Reference v3 (No. 10) .......... .......... .......... .....T.... .......... .......... ...A...... 490
v3 (n=1)             .......... .......... .......... .....T.... .......... .......... ...A...... 490
Reference v4 (93b_S9) .......... .......... .......... .......... .......... .......... ...A...... 490
v4 (n=1)             .......... .......... .......... .......... .......... .......... ...A...... 490
v5 (RKI4, SAI33)     .......... .......... .......... .....T.... .......... .......... ...A...... 490

                               500                   520                   540                   560
Reference v1 (COL)   TTGAAGATGG TAAAAATTTA TTATCTTTTG ACGTACAAAC TAATAAGAAA AAGGTGACTG CTCAAGAATT 560
v1 (n=5)             .......... .......... .......... .......... .......... .......... .......... 560
Reference v2 (IVM10) .......... .......... .......... .......... .......... ..A....... .......... 560
v2 (n=1)             .......... .......... .......... .......... .......... ..A....... .......... 560
Reference v3 (No. 10) .......... .......... ..G....... .......... .......... ..A....... .......... 560
v3 (n=1)             .......... .......... ..G....... .......... .......... ..A....... .......... 560
Reference v4 (93b_S9) .......... .......... .......... .......... .......... ..A....... .......... 560
v4 (n=1)             .......... .......... .......... .......... .......... ..A....... .......... 560
v5 (RKI4, SAI33)     .......... .......... ..G....... .......... .......... ..A....... .......... 560
```

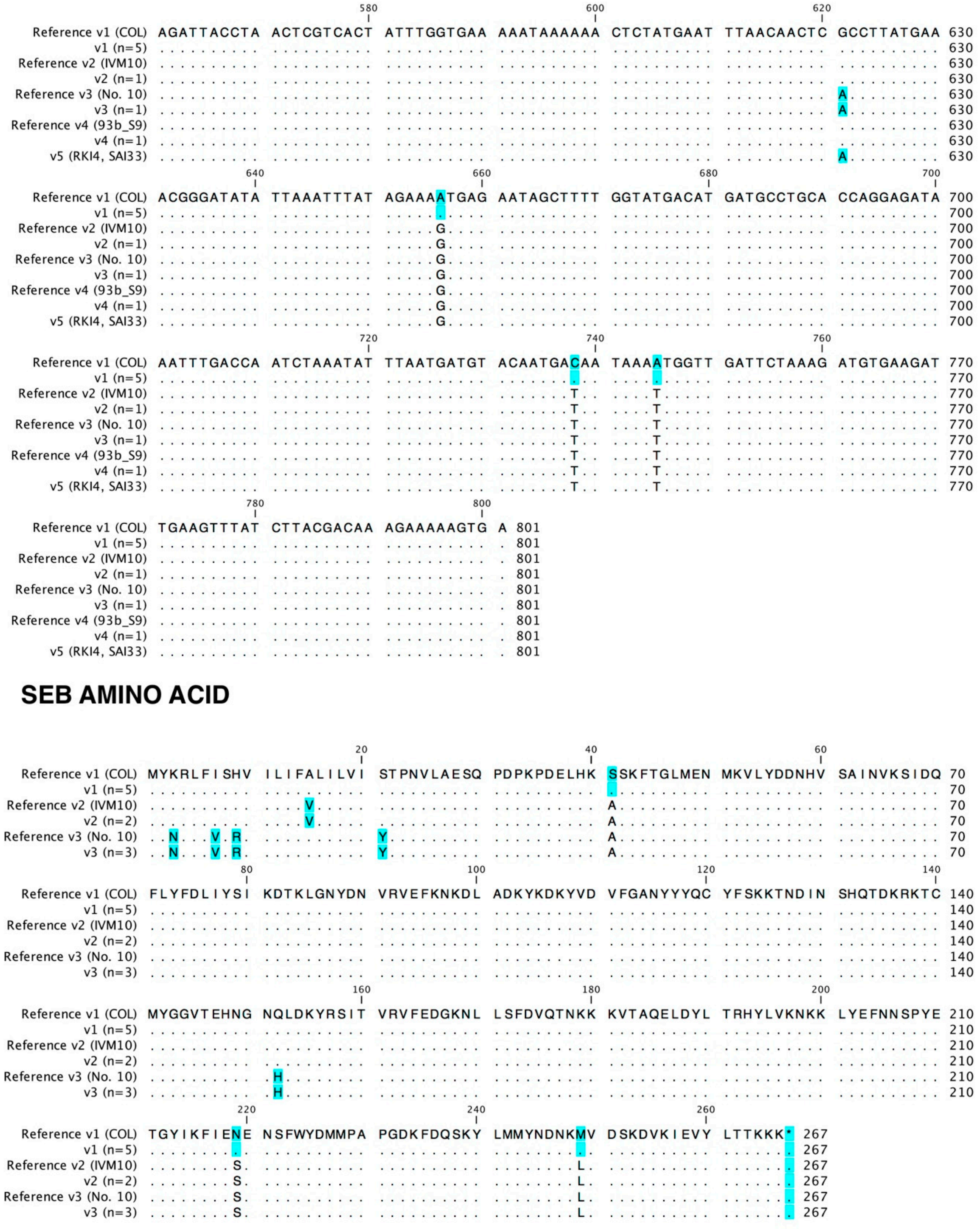

Figure S1. Sequence alignments for *seb*.

SEC PROMOTER

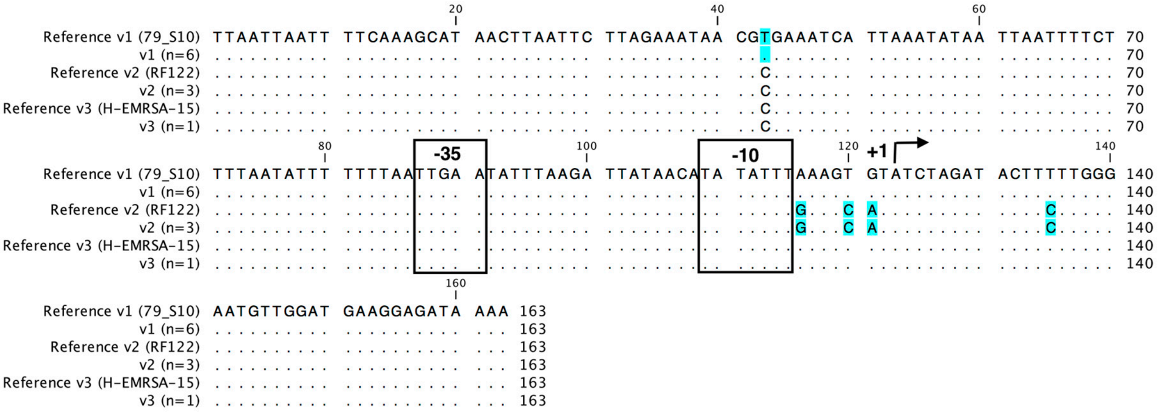

SEC GENE

```
                                                20                    40                    60
                                                 |                     |                     |
Reference v1 (sec-2)     ATGAATAAGA GTCGATTTAT TTCATGCGTA ATTTTGATAT TCGCACTTAT ACTAGTTCTT TTTACACCCA 70
v1 (n=5)                 .......... .......... .......... .......... .......... .......... .......... 70
Reference v2 (sec-bovine) .......... .......... .......... .......... .......... .......... .......... 70
v2 (n=3)                 .......... .......... .......... .......... .......... .......... .......... 70
Reference v3 (sec-1)     .......... .......... .......... .......... .......... .......... .......... 70
v3 (n=1)                 .......... .......... .......... .......... .......... .......... .......... 70
v4 (SAI48)               .......... .......... .......... .......... .......... .......... .......... 70
Reference (sec-3)        ...T...... .ATT...... ....C.T... .......... .......G.. .......A.. .C........ 70
Reference (sec-4)        .......... .......... .......... .......... .......... .......... .......... 70
Reference (sec-ovine)    .......... .......... .......... .......... ..T....... .......... .......... 70

                                   80                   100                   120                   140
                                    |                     |                     |                     |
Reference v1 (sec-2)     ACGTATTAGC AGAGAGCCAA CCAGACCCTA CGCCAGATGA GTTGCACAAA TCAAGTGAGT TTACTGGTAC 140
v1 (n=5)                 .......... .......... .......... .......... .......... .......... .......... 140
Reference v2 (sec-bovine) .......... .......... .......... .......... .......... G.G...A.A. .C......TT 140
v2 (n=3)                 .......... .......... .......... .......... .......... G.G...A.A. .C......TT 140
Reference v3 (sec-1)     .......... .......... .......... .......... .......... G.G...A.A. .C......TT 140
v3 (n=1)                 .......... .......... .......... .......... .......... G.G...A.A. .C......TT 140
v4 (SAI48)               .......... .......... .......... .......... .......... .......... .......... 140
Reference (sec-3)        ....T..... ......T... .......... T......... T......... .......... .......... 140
Reference (sec-4)        .......... .......... .......... .......... .......... .......... .......... 140
Reference (sec-ovine)    .......... .......... .......... .......... .......... G.G...A.A. .C......TT 140

                                              160                   180                   200
                                                |                     |                     |
Reference v1 (sec-2)     GATGGGTAAT ATGAAATATT TATATGATGA TCATTATGTA TCAGCAACTA AAGTTATGTC TGTAGATAAA 210
v1 (n=5)                 .......... .......... .......... .......... .......... .......... .......... 210
Reference v2 (sec-bovine) .....AA... ......GT.. .......... ..G....... .......... ......A... .......... 210
v2 (n=3)                 .....AA... ......GT.. .......... ..G....... .......... ......A... .......... 210
Reference v3 (sec-1)     .....AA... ......GT.. .......... .......... .......... ......A... .......... 210
v3 (n=1)                 .....AA... ......GT.. .......... .......... .......... ......A... .......... 210
v4 (SAI48)               .......... .......... .......... .......... .......... .......... .......... 210
Reference (sec-3)        .......... .......... .......... .......... .......... ......A... .......... 210
Reference (sec-4)        .......... .......... .......... .......... .......... ......A... .......... 210
Reference (sec-ovine)    .....AA... ......GT.. .......... ..G....... .......... ......A... .......... 210

                                  220                   240                   260                   280
                                    |                     |                     |                     |
Reference v1 (sec-2)     TTTTTGGCAC ATGATTTAAT TTATAACATT AGTGATAAAA AACTAAAAAA TTATGACAAA GTGAAAACAG 280
v1 (n=5)                 .......... .......... .......... .......... .......... .......... .......... 280
Reference v2 (sec-bovine) .......... .......... .......... .......... ....G..... .......... .......... 280
v2 (n=3)                 .......... .......... .......... .......... ....G..... .......... .......... 280
Reference v3 (sec-1)     .......... .......... .......... .......... ....G..... .......... .......... 280
v3 (n=1)                 .......... .......... .......... .......... ....G..... .......... .......... 280
v4 (SAI48)               .......... .......... .......... .......... .......... .......... .......... 280
Reference (sec-3)        .......... .......... .......... .......... ....G..... .......... .......... 280
Reference (sec-4)        .......... .......... .......... .......... .......... .......... .......... 280
Reference (sec-ovine)    .......... .......... .......... .......... ....G..... .......... .......... 280

                                              300                   320                   340
                                                |                     |                     |
Reference v1 (sec-2)     AGTTATTAAA TGAAGATTTA GCAAAGAAGT ACAAAGATGA AGTAGTTGAT GTGTATGGAT CAAATTACTA 350
v1 (n=5)                 .......... .......... .......... .......... .......... .......... .......... 350
Reference v2 (sec-bovine) .......... .......... .......... .......... .......... .......... .......... 350
v2 (n=3)                 .......... .......... .......... .......... .......... .......... .......... 350
Reference v3 (sec-1)     .......... .....G.... .......... .......... .......... .......... .......... 350
v3 (n=1)                 .......... .....G.... .......... .......... .......... .......... .......... 350
v4 (SAI48)               .......... .......... .......... .......... .......... .......... .......... 350
Reference (sec-3)        .......... .......... .......... .......... .......... .......... .......... 350
Reference (sec-4)        .......... .......... .....A.... .......... .......... .......... .......... 350
Reference (sec-ovine)    .......... .......... .......... .......... .......... .......... .......... 350

                                  360                   380                   400                   420
                                    |                     |                     |                     |
Reference v1 (sec-2)     TGTAAACTGC TATTTTTCAT CCAAAGATAA TGTAGGTAAA GTTACAGGTG GTAAAACTTG TATGTATGGA 420
v1 (n=5)                 .......... .......... .......... .......... .......... .......... .......... 420
Reference v2 (sec-bovine) .......... .......... .......... .......... .......... .......... .......... 420
v2 (n=3)                 .......... .......... .......... .......... .......... .......... .......... 420
Reference v3 (sec-1)     .......... .......... .......... .......... .......... .C........ .......... 420
v3 (n=1)                 .......... .......... .......... .......... .......... .C........ .......... 420
v4 (SAI48)               .......... .......... .......... .......... .......... .......... .......... 420
Reference (sec-3)        .......... .......... .......... .......... .......... .......... .......... 420
Reference (sec-4)        .......... .......... .......... .......... .......... .......... .......... 420
Reference (sec-ovine)    .......... .G........ .......... .......... .......... .......... .......... 420

                                              440                   460                   480
                                                |                     |                     |
Reference v1 (sec-2)     GGAATAACAA AACATGAAGG AAACCACTTT GATAATGGGA ACTTACAAAA TGTACTTATA AGAGTTTATG 490
v1 (n=5)                 .......... .......... .......... .......... .......... .......... .......... 490
Reference v2 (sec-bovine) .......... .......... .......... .......... .A........ .......... .......... 490
v2 (n=3)                 .......... .......... .......... .......... .A........ .......... .......... 490
Reference v3 (sec-1)     .......... .......... .......... .......... .......... .......... .......... 490
v3 (n=1)                 .......... .......... .......... .......... .......... .......... .......... 490
v4 (SAI48)               .......... .......... .......... .......... .......... .......... .......... 490
Reference (sec-3)        .......... .......... .......... .......... .......... .......G.. .......... 490
Reference (sec-4)        .......... .......... .......... .......... .......... .......... .......... 490
Reference (sec-ovine)    .......... .......... .......... .......... .......... .......... .......... 490

                                  500                   520                   540                   560
                                    |                     |                     |                     |
Reference v1 (sec-2)     AAAATAAAAG AACACAATTT TCTTTTGAAG TGCAAACTGA TAAGAAAAGT GTAACAGCTC AAGAACTAGA 560
v1 (n=5)                 .......... .......... .......... .......... .......... .......... .......... 560
Reference v2 (sec-bovine) .......... .......... .......... .......... .......... .......... .......... 560
v2 (n=3)                 .......... .......... .......... .......... .......... .......... .......... 560
Reference v3 (sec-1)     .......... .......... .......... .......... .......... .......... .......... 560
v3 (n=1)                 .......... .......... .......... .......... .......... .......... .......... 560
v4 (SAI48)               .......... .......... .......... .......... .......... .......... .......... 560
Reference (sec-3)        .......... .......... .......... .......... .......... .......... .......... 560
Reference (sec-4)        .......... .......... .......... .......... .......... .......... .......... 560
Reference (sec-ovine)    .......... .......... .......... .......... .......... .......... .......... 560
```

```
                                           580                          600                          620
                            |                            |                            |
Reference v1 (sec-2)  CATAAAAGCT AGGAATTTTT TAATTAATAA AAAAAATTTG TATGAGTTTA ACAGTTCACC ATATGAAACA 630
            v1 (n=5)  .......... .......... .......... .......... .......... .......... .......... 630
Reference v2 (sec-bovine)  .......... .......... .......... .......... .......... .......... .......... 630
            v2 (n=3)  .......... .......... .......... .......... .......... .......... .......... 630
Reference v3 (sec-1)  .......... .......... .......... .......... .......... .......... .......... 630
            v3 (n=1)  .......... .......... .......... .......... .......... .......... .......... 630
          v4 (SAI48)  .......... .......... .......... .......... .......... .......... .......... 630
  Reference (sec-3)   .......... .......... .......... .......... .......... .......... .......... 630
  Reference (sec-4)   .......... .......... .......... .......... .......... .......... .......... 630
Reference (sec-ovine) .......... ....G..... .......... .......... .......... .......... .......... 630

                 640                          660                          680                          700
Reference v1 (sec-2)  GGATATATAA AATTTATTGA AAATAACGGC AATACTTTTT GGTATGATAT GATGCCTGCA CCAGGCGATA 700
            v1 (n=5)  .......... .......... .......... .......... .......... .......... .......... 700
Reference v2 (sec-bovine)  ..G....... .......... .......... .......... .......... .......... .....A.... 700
            v2 (n=3)  ..G....... .......... .......... .......... .......... .......... .....A.... 700
Reference v3 (sec-1)  .......... .......... .......... .......... .......... .......... .......... 700
            v3 (n=1)  .......... .......... .......... .......... .......... .......... .......... 700
          v4 (SAI48)  .......... .......... .......... .......... .......... .......... .......... 700
  Reference (sec-3)   .......... .......... .......... .......... .......... .......... .......... 700
  Reference (sec-4)   .......... .......... .......... .......... .......... .......... .......... 700
Reference (sec-ovine) .......... .......... .......... .......... .......... .......... .....A.... 700

                                           720                          740                          760
Reference v1 (sec-2)  AGTTTGACCA ATCTAAATAT TTAATGATGT ACAACGACAA TAAAACGGTT GATTCTAAAA GTGTGAAGAT 770
            v1 (n=5)  .......... .......... .......... .......... .......... .......... .......... 770
Reference v2 (sec-bovine)  .......... .......... .......... .......... .......... .......... .......... 770
            v2 (n=3)  .......... .......... .......... .......... .......... .......... .......... 770
Reference v3 (sec-1)  .......... .......... .......... .......... .......... .......... .......... 770
            v3 (n=1)  .......... .......... .......... .......... .......... .......... .......... 770
          v4 (SAI48)  .......... .......... .......... .......... ......A... .......... .......... 770
  Reference (sec-3)   .......... .......... .......... .......... .......... .......... .......... 770
  Reference (sec-4)   .......... .......... .......... .......... .......... .......... .A........ 770
Reference (sec-ovine) .......... .......... .......... .......... .......... .......... .......... 770

                 780                          800
Reference v1 (sec-2)  AGAAGTCCAC CTTACAACAA AGAATGGATA A 801
            v1 (n=5)  .......... .......... .......... . 801
Reference v2 (sec-bovine)  .......... .......... .......... . 801
            v2 (n=3)  .......... .......... .......... . 801
Reference v3 (sec-1)  .......... .......... .......... . 801
            v3 (n=1)  .......... .......... .......... . 801
          v4 (SAI48)  .......... .......... .......... . 801
  Reference (sec-3)   .......... .......... .......... . 801
  Reference (sec-4)   .......... .......... .......... . 801
Reference (sec-ovine) .......... .......... .......... . 801
```

SEC AMINO ACID

```
                                  20                           40                           60
Reference v1 (sec-2)  MNKSRFISCV ILIFALILVL FTPNVLAESQ PDPTPDELHK SSEFTGTMGN MKYLYDDHYV SATKVMSVDK 70
            v1 (n=6)  .......... .......... .......... .......... .......... .......... .......... 70
Reference v2 (sec-bovine)  .......... .......... .......... .......... A.K...L.E. ..V....R.. .....K.... 70
            v2 (n=3)  .......... .......... .......... .......... A.K...L.E. ..V....R.. .....K.... 70
Reference v3 (sec-1)  .......... .......... .......... .......... A.K...L.E. ..V....... .....K.... 70
            v3 (n=1)  .......... .......... .......... .......... A.K...L.E. ..V....... .....K.... 70
  Reference (sec-3)   .Y.RL...R. .........I S......... ...M..D... .......... .......... .....K.... 70
  Reference (sec-4)   .......... .......... .......... .......... .......... .......... .....K.... 70
Reference (sec-ovine) .......... ....S..... .......... .......... A.K...L.E. ..V....R.. .....K.... 70

                 80                           100                          120                          140
Reference v1 (sec-2)  FLAHDLIYNI SDKKLKNYDK VKTELLNEDL AKKYKDEVVD VYGSNYYVNC YFSSKDNVGK VTGGKTCMYG 140
            v1 (n=6)  .......... .......... .......... .......... .......... .......... .......... 140
Reference v2 (sec-bovine)  .......... .......... .......... .......... .......... .......... .......... 140
            v2 (n=3)  .......... .......... .......... .......... .......... .......... .......... 140
Reference v3 (sec-1)  .......... .......... ........G. .......... .......... .......... .......... 140
            v3 (n=1)  .......... .......... ........G. .......... .......... .......... .......... 140
  Reference (sec-3)   .......... .......... .......... .......... .......... .......... .......... 140
  Reference (sec-4)   .......... .......... .......... .......... .......... .......... .......... 140
Reference (sec-ovine) .......... .......... .......... .......... .......... C......... .......... 140

                                  160                          180                          200
Reference v1 (sec-2)  GITKHEGNHF DNGNLQNVLI RVYENKRNTI SFEVQTDKKS VTAQELDIKA RNFLINKKNL YEFNSSPYET 210
            v1 (n=6)  .......... .......... .......... .......... .......... .......... .......... 210
Reference v2 (sec-bovine)  .......... ...K...... .......... .......... .......... .......... .......... 210
            v2 (n=3)  .......... ...K...... .......... .......... .......... .......... .......... 210
Reference v3 (sec-1)  .......... .......... .......... .......... .......... .......... .......... 210
            v3 (n=1)  .......... .......... .......... .......... .......... .......... .......... 210
  Reference (sec-3)   .......... .........V .......... .......... .......... .......... .......... 210
  Reference (sec-4)   .......... .......... .......... .......... .......... .......... .......... 210
Reference (sec-ovine) .......... .......... .......... .......... .......... .S........ .......... 210

                 220                          240                          260
Reference v1 (sec-2)  GYIKFIENNG NTFWYDMMPA PGDKFDQSKY LMMYNDNKTV DSKSVKIEVH LTTKNG* 267
            v1 (n=6)  .......... .......... .......... .......... .......... ....... 267
Reference v2 (sec-bovine)  .......... .......... .......... .......... .......... ....... 267
            v2 (n=3)  .......... .......... .......... .......... .......... ....... 267
Reference v3 (sec-1)  .......... .......... .......... .......... .......... ....... 267
            v3 (n=1)  .......... .......... .......... .......... .......... ....... 267
  Reference (sec-3)   .......... .......... .......... .......... .......... ......- 266
  Reference (sec-4)   .......... .......... .......... .......... ...R...... ....... 267
Reference (sec-ovine) .......... .......... .......... .......... .......... ....... 267
```

Figure S2. Sequence alignments for *sec*.

SED PROMOTER

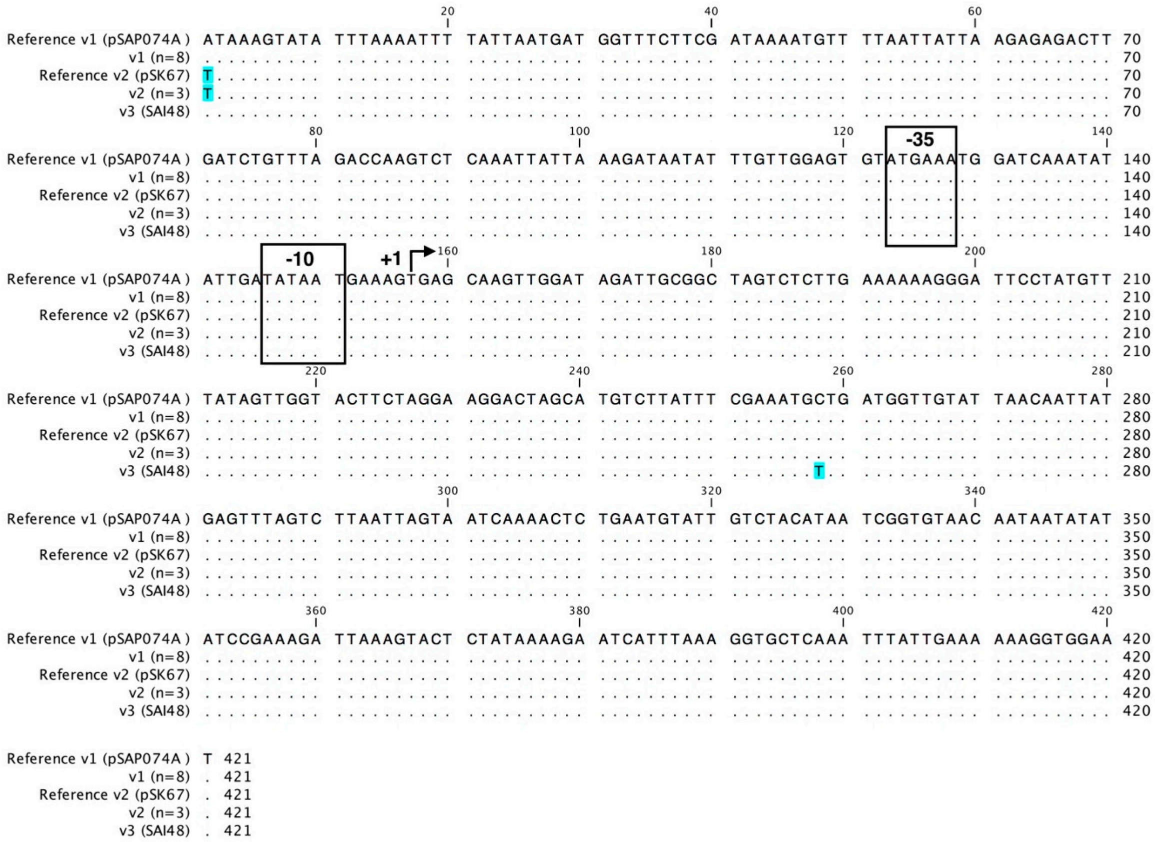

SED GENE

```
                                           20                  40                  60
                                            |                   |                   |
Reference v1 (pSAP074A) ATGAAAAAAT TTAACATTCT TATTGCATTA CTCTTTTTTA CTAGTTTGGT AATATCTCCT TTAAACGTTA 70
              v1 (n=4)  .......... .......... .......... .......... .......... .......... .......... 70
   Reference v2 (pSK67) .......... .......... .......... .......... .......... .......... .......... 70
              v2 (n=4)  .......... .......... .......... .......... .......... .......... .......... 70
  Reference v3 (P502A)  .......... .......... .......... .......... .......... .......... .......... 70
              v3 (n=6)  .......... .......... .......... .......... .......... .......... .......... 70
          v4 (SANC30)   .......... .......... .......... .......... .......... .......... .......... 70
    Reference (pIB485)  .......... .......... .......... .......... .......... .......... .......... 70
                                80                 100                 120                 140
                                 |                   |                   |                   |
Reference v1 (pSAP074A) AAGCCAATGA AAACATTGAT TCAGTAAAAG AGAAAGAATT GCATAAAAAA TCTGAATTAA GTAGTACCGC 140
              v1 (n=4)  .......... .......... .......... .......... .......... .......... .......... 140
   Reference v2 (pSK67) .......... .......... .......... .......... .......... .......... .......... 140
              v2 (n=4)  .......... .......... .......... .......... .......... .......... .......... 140
  Reference v3 (P502A)  .......... .......... .......... .......... .......... .......... .......... 140
              v3 (n=6)  .......... .......... .......... .......... .......... .......... .......... 140
          v4 (SANC30)   .......... .......... .......... .......... .......... .......... .......... 140
    Reference (pIB485)  .......... .......... .......... .......... .......... .......... .......... 140
                                          160                 180                 200
                                            |                   |                   |
Reference v1 (pSAP074A) GCTAAATAAT ATGAAACATT CTTATGCAGA TAAAAATCCA ATAATAGGAG AAAATAAAAG TACAGGAGAT 210
              v1 (n=4)  .......... .......... .......... .......... .......... .......... .......... 210
   Reference v2 (pSK67) .......... .......... .......... .......... .......... .......... .......... 210
              v2 (n=4)  .......... .......... .......... .......... .......... .......... .......... 210
  Reference v3 (P502A)  .......... .......... .......... .......... .......... .......... .......... 210
              v3 (n=6)  .......... .......... .......... .......... .......... .......... .......... 210
          v4 (SANC30)   .......... .......... .......... .......... .......... .......... .......... 210
    Reference (pIB485)  .......... .......... .......... .......... .......... .......... .......... 210
                               220                 240                 260                 280
                                 |                   |                   |                   |
Reference v1 (pSAP074A) CAATTTTTAG AAAATACTTT GCTTTACAAA AAATTTTTTA CTGACCTTAT CAATTTTGAA GATTTATTAA 280
              v1 (n=4)  .......... .......... .......... .......... .......... .......... .......... 280
   Reference v2 (pSK67) .......... .......... .......... .......... .......... .......... .......... 280
              v2 (n=4)  .......... .......... .......... .......... .......... .......... .......... 280
  Reference v3 (P502A)  .......... .......... .......... .......... .......... .......... .......... 280
              v3 (n=6)  .......... .......... .......... .......... .......... .......... .......... 280
          v4 (SANC30)   .......... .......... .......... .......... .......... .......... .......... 280
    Reference (pIB485)  .......... .......... .......... .......... .......... .......... .......... 280
                                          300                 320                 340
                                            |                   |                   |
Reference v1 (pSAP074A) TAAACTTCAA TTCAAAAGAA ATGGCTCAAC ATTTCAAATC TAAAAATGTA GATGTTTACG CTATAAGATA 350
              v1 (n=4)  .......... .......... .......... .......... .......... .......... .......... 350
   Reference v2 (pSK67) .......... .......... .......... .......... .......... .......... .......... 350
              v2 (n=4)  .......... .......... .......... .......... .......... .......... .......... 350
  Reference v3 (P502A)  .......... .......... .......... .......... .......... .......... .......... 350
              v3 (n=6)  .......... .......... .......... .......... .......... .......... .......... 350
          v4 (SANC30)   .......... .......A.. .......... .......... .......... .......... .......... 350
    Reference (pIB485)  .......... .......... .......... .......... .......... .........C .......... 350
                               360                 380                 400                 420
                                 |                   |                   |                   |
Reference v1 (pSAP074A) TAGCATTAAT TGTTATGGTG GTGAAATAGA TAGGACTGCT TGTACATATG GAGGTGTCAC TCCACACGAA 420
              v1 (n=4)  .......... .......... .......... .......... .......... .......... .......... 420
   Reference v2 (pSK67) .......... .......... .......... ..A....... .......... .......... .......... 420
              v2 (n=4)  .......... .......... .......... ..A....... .......... .......... .......... 420
  Reference v3 (P502A)  .......... .......... .......... .......... .......... .......... .......... 420
              v3 (n=6)  .......... .......... .......... .......... .......... .......... .......... 420
          v4 (SANC30)   .......... ...G...... .......... .......... .......... .......... .......... 420
    Reference (pIB485)  .......... .......... .......... .......... .......... .......... .......... 420
                                          440                 460                 480
                                            |                   |                   |
Reference v1 (pSAP074A) GGTAATAAAT TAAAAGAACG AAAAAAAATA CCAATCAATT TGTGGATAAA TGGTGTACAA AAAGAAGTTT 490
              v1 (n=4)  .......... .......... .......... .......... .......... .......... .......... 490
   Reference v2 (pSK67) .......... .......... .......... .......... .......... .......... .......... 490
              v2 (n=4)  .......... .......... .......... .......... .......... .......... .......... 490
  Reference v3 (P502A)  .......... .......... .......... .......... .......... .......... .......... 490
              v3 (n=6)  .......... .......... .......... .......... .......... .......... .......... 490
          v4 (SANC30)   .......... .......... .......... .......... .......... .......... .......... 490
    Reference (pIB485)  .......... .......... .......... .......... .......... .......... .......... 490
                               500                 520                 540                 560
                                 |                   |                   |                   |
Reference v1 (pSAP074A) CTTTAGATAA AGTTCAAACA GATAAAAAAA ATGTTACCGT ACAAGAATTA GATGCACAAG CAAGGCGCTA 560
              v1 (n=4)  .......... .......... .......... .......... .......... .......... .......... 560
   Reference v2 (pSK67) .......... .......... .......... .......... .......... .......... .......... 560
              v2 (n=4)  .......... .......... .......... .......... .......... .......... .......... 560
  Reference v3 (P502A)  .......... .......... .......... -......... .......... .......... .......... 559
              v3 (n=6)  .......... .......... .......... -......... .......... .......... .......... 559
          v4 (SANC30)   .......... .......... .......... .......... .......... .......... .......... 560
    Reference (pIB485)  .......... .......... .......... .......... .......... .......... .......... 560
```

```
                                                580                                    600                                    620
                                                 |                                      |                                      |
Reference v1 (pSAP074A)  TTTGCAAAAG  GATTTAAAAT  TGTATAATAA  TGATACTCTC  GGAGGAAAAA  TACAGCGCGG  AAAAATAGAG  630
               v1 (n=4)  . . . . . . . . . .  . . . . . . . . . .  . . . . . . . . . .  . . . . . . . . . .  . . . . . . . . . .  . . . . . . . . . .  . . . . . . . . . .  630
  Reference v2 (pSK67)  . . . . . . . . . .  . . . . . . . . . .  . . . . . . . . . .  . . . . . . . . . .  . . . . . . . . . .  . . . . . . . . . .  . . . . . . . . . .  630
               v2 (n=4)  . . . . . . . . . .  . . . . . . . . . .  . . . . . . . . . .  . . . . . . . . . .  . . . . . . . . . .  . . . . . . . . . .  . . . . . . . . . .  630
  Reference v3 (P502A)  . . . . . . . . . .  . . . . . . . . . .  . . . . . . . . . .  . . . . . . . . . .  . . . . . . . . . .  . . . . . . . . . .  . . . . . . . . . .  629
               v3 (n=6)  . . . . . . . . . .  . . . . . . . . . .  . . . . . . . . . .  . . . . . . . . . .  . . . . . . . . . .  . . . . . . . . . .  . . . . . . . . . .  629
            v4 (SANC30)  . . . . . . . . . .  . . . . . . . . . .  . . . . . . . . . .  . . . . . . . . . .  . . . . . . . . . .  . . . . . . . . . .  . . . . . . . . . .  630
      Reference (pIB485)  . . . . . . . . . .  . . . . . . . . . .  . . . . . . . . . .  . . . . . . . . . .  . . . . . . . . . .  . . . . . . . . . .  . . . . . . . . . .  630

                     640                                    660                                    680                                    700
                      |                                      |                                      |                                      |
Reference v1 (pSAP074A)  TTTGATTCTT  CTGATGGGTC  TAAAGTCTCT  TATGATTTAT  TTGATGTTAA  GGGTGATTTT  CCCGAAAAAC  700
               v1 (n=4)  . . . . . . . . . .  . . . . . . . . . .  . . . . . . . . . .  . . . . . . . . . .  . . . . . . . . . .  . . . . . . . . . .  . . . . . . . . . .  700
  Reference v2 (pSK67)  . . . . . . . . . .  . . . . . . . . . .  . . . . . . . . . .  . . . . . . . . . .  . . . . . . . . . .  . . . . . . . . . .  . . . . . . . . . .  700
               v2 (n=4)  . . . . . . . . . .  . . . . . . . . . .  . . . . . . . . . .  . . . . . . . . . .  . . . . . . . . . .  . . . . . . . . . .  . . . . . . . . . .  700
  Reference v3 (P502A)  . . . . . . . . . .  . . . . . . . . . .  . . . . . . . . . .  . . . . . . . . . .  . . . . . . . . . .  . . . . . . . . . .  . . . . . . . . . .  699
               v3 (n=6)  . . . . . . . . . .  . . . . . . . . . .  . . . . . . . . . .  . . . . . . . . . .  . . . . . . . . . .  . . . . . . . . . .  . . . . . . . . . .  699
            v4 (SANC30)  . . . . . . . . . .  . . . . . . . . . .  . . . . . . . . . .  . . . . . . . . . .  . . . . . . . . . .  . . . . . . . . . .  . . . . . . . . . .  700
      Reference (pIB485)  . . . . . . . . . .  . . . . . . . . . .  . . . . . . . . . .  . . . . . . . . . .  . . . . . . . . . .  . . . . . . . . . .  . . . . . . . . . .  700

                                          720                                    740                                    760
                                           |                                      |                                      |
Reference v1 (pSAP074A)  AATTACGAAT  ATACAGTGAT  AATAAAACAT  TATCCACAGA  GCACCTTCAT  ATTGACATCT  ATTTATATGA  770
               v1 (n=4)  . . . . . . . . . .  . . . . . . . . . .  . . . . . . . . . .  . . . . . . . . . .  . . . . . . . . . .  . . . . . . . . . .  . . . . . . . . . .  770
  Reference v2 (pSK67)  . . . . . . . . . .  . . . . . . . . . .  . . . . . . . . . .  . . . . . . . . . .  . . . . . . . . . .  . . . . . . . . . .  . . . . . . . . . .  770
               v2 (n=4)  . . . . . . . . . .  . . . . . . . . . .  . . . . . . . . . .  . . . . . . . . . .  . . . . . . . . . .  . . . . . . . . . .  . . . . . . . . . .  770
  Reference v3 (P502A)  . . . . . . . . . .  . . . . . . . . . .  . . . . . . . . . .  . . . . . . . . . .  . . . . . . . . . .  . . . . . . . . . .  . . . . . . . . . .  769
               v3 (n=6)  . . . . . . . . . .  . . . . . . . . . .  . . . . . . . . . .  . . . . . . . . . .  . . . . . . . . . .  . . . . . . . . . .  . . . . . . . . . .  769
            v4 (SANC30)  . . . . . . . . . .  . . . . . . . . . .  . . . . . . . . . .  . . . . . . . . . .  . . . . . . . . . .  . . . . . . . . . .  . . . . . . . . . .  770
      Reference (pIB485)  . . . . . . . . . .  . . . . . . . . . .  . . . . . . . . . .  . . . . . . . . . .  . . . . . . . . . .  . . . . . . . . . .  . . . . . . . . . .  770

Reference v1 (pSAP074A)  AAAGTAG  777
               v1 (n=4)  . . . . . . .  777
  Reference v2 (pSK67)  . . . . . . .  777
               v2 (n=4)  . . . . . . .  777
  Reference v3 (P502A)  . . . . . . .  776
               v3 (n=6)  . . . . . . .  776
            v4 (SANC30)  . . . . . . .  777
      Reference (pIB485)  . . . . . . .  777
```

SED AMINO ACID

```
                                          20                                     40                                     60
                                           |                                      |                                      |
Reference v1 (pSAP074A)  MKKFNILIAL  LFFTSLVISP  LNVKANENID  SVKEKELHKK  SELSSTALNN  MKHSYADKNP  IIGENKSTGD  70
               v1 (n=4)  . . . . . . . . . .  . . . . . . . . . .  . . . . . . . . . .  . . . . . . . . . .  . . . . . . . . . .  . . . . . . . . . .  . . . . . . . . . .  70
  Reference v2 (pSK67)  . . . . . . . . . .  . . . . . . . . . .  . . . . . . . . . .  . . . . . . . . . .  . . . . . . . . . .  . . . . . . . . . .  . . . . . . . . . .  70
               v2 (n=4)  . . . . . . . . . .  . . . . . . . . . .  . . . . . . . . . .  . . . . . . . . . .  . . . . . . . . . .  . . . . . . . . . .  . . . . . . . . . .  70
  Reference v3 (p502A)  . . . . . . . . . .  . . . . . . . . . .  . . . . . . . . . .  . . . . . . . . . .  . . . . . . . . . .  . . . . . . . . . .  . . . . . . . . . .  70
               v3 (n=6)  . . . . . . . . . .  . . . . . . . . . .  . . . . . . . . . .  . . . . . . . . . .  . . . . . . . . . .  . . . . . . . . . .  . . . . . . . . . .  70
            v4 (SANC30)  . . . . . . . . . .  . . . . . . . . . .  . . . . . . . . . .  . . . . . . . . . .  . . . . . . . . . .  . . . . . . . . . .  . . . . . . . . . .  70
      Reference (pIB485)  . . . . . . . . . .  . . . . . . . . . .  . . . . . . . . . .  . . . . . . . . . .  . . . . . . . . . .  . . . . . . . . . .  . . . . . . . . . .  70

                     80                                    100                                    120                                    140
                      |                                      |                                      |                                      |
Reference v1 (pSAP074A)  QFLENTLLYK  KFFTDLINFE  DLLINFNSKE  MAQHFKSKNV  DVYAIRYSIN  CYGGEIDRTA  CTYGGVTPHE  140
               v1 (n=4)  . . . . . . . . . .  . . . . . . . . . .  . . . . . . . . . .  . . . . . . . . . .  . . . . . . . . . .  . . . . . . . . . .  . . . . . . . . . .  140
  Reference v2 (pSK67)  . . . . . . . . . .  . . . . . . . . . .  . . . . . . . . . .  . . . . . . . . . .  . . . . . . . . . .  . . . . . . . K . .  . . . . . . . . . .  140
               v2 (n=4)  . . . . . . . . . .  . . . . . . . . . .  . . . . . . . . . .  . . . . . . . . . .  . . . . . . . . . .  . . . . . . . K . .  . . . . . . . . . .  140
  Reference v3 (p502A)  . . . . . . . . . .  . . . . . . . . . .  . . . . . . . . . .  . . . . . . . . . .  . . . . . . . . . .  . . . . . . . . . .  . . . . . . . . . .  140
               v3 (n=6)  . . . . . . . . . .  . . . . . . . . . .  . . . . . . . . . .  . . . . . . . . . .  . . . . . . . . . .  . . . . . . . . . .  . . . . . . . . . .  140
            v4 (SANC30)  . . . . . . . . . .  . . . . . . . . . .  . . . . . . . . . K  . . . . . . . . . .  . . . . . . . . . .  . D . . . . . . . .  . . . . . . . . . .  140
      Reference (pIB485)  . . . . . . . . . .  . . . . . . . . . .  . . . . . . . . . .  . . . . . . . . . .  . . . P . . . . . .  . . . . . . . . . .  . . . . . . . . . .  140

                                          160                                    180                                    200
                                           |                                      |                                      |
Reference v1 (pSAP074A)  GNKLKERKKI  PINLWINGVQ  KEVSLDKVQT  DKKNV----T  VQELDAQARR  YLQKDLKLYN  NDTLGGKIQR  206
               v1 (n=4)  . . . . . . . . . .  . . . . . . . . . .  . . . . . . . . . .  . . . . . - - - - .  . . . . . . . . . .  . . . . . . . . . .  . . . . . . . . . .  206
  Reference v2 (pSK67)  . . . . . . . . . .  . . . . . . . . . .  . . . . . . . . . .  . . . . . - - - - .  . . . . . . . . . .  . . . . . . . . . .  . . . . . . . . . .  206
               v2 (n=4)  . . . . . . . . . .  . . . . . . . . . .  . . . . . . . . . .  . . . . . - - - - .  . . . . . . . . . .  . . . . . . . . . .  . . . . . . . . . .  206
  Reference v3 (p502A)  . . . . . . . . . .  . . . . . . . . . .  . . . . . . . . . .  . . . MLPYKN*  ----------  ----------  ----------  180
               v3 (n=6)  . . . . . . . . . .  . . . . . . . . . .  . . . . . . . . . .  . . . MLPYKN*  ----------  ----------  ----------  180
            v4 (SANC30)  . . . . . . . . . .  . . . . . . . . . .  . . . . . . . . . .  . . . . . - - - - .  . . . . . . . . . .  . . . . . . . . . .  . . . . . . . . . .  206
      Reference (pIB485)  . . . . . . . . . .  . . . . . . . . . .  . . . . . . . . . .  . . . . . - - - - .  . . . . . . . . . .  . . . . . . . . . .  . . . . . . . . . .  206

                     220                                    240                                    260
                      |                                      |                                      |
Reference v1 (pSAP074A)  GKIEFDSSDG  SKVSYDLFDV  KGDFPEKQLR  IYSDNKTLST  EHLHIDIYLY  EK*  259
               v1 (n=4)  . . . . . . . . . .  . . . . . . . . . .  . . . . . . . . . .  . . . . . . . . . .  . . . . . . . . . .  . . .  259
  Reference v2 (pSK67)  . . . . . . . . . .  . . . . . . . . . .  . . . . . . . . . .  . . . . . . . . . .  . . . . . . . . . .  . . .  259
               v2 (n=4)  . . . . . . . . . .  . . . . . . . . . .  . . . . . . . . . .  . . . . . . . . . .  . . . . . . . . . .  . . .  259
  Reference v3 (p502A)  ----------  ----------  ----------  ----------  ----------  ---  180
               v3 (n=6)  ----------  ----------  ----------  ----------  ----------  ---  180
            v4 (SANC30)  . . . . . . . . . .  . . . . . . . . . .  . . . . . . . . . .  . . . . . . . . . .  . . . . . . . . . .  . . .  259
      Reference (pIB485)  . . . . . . . . . .  . . . . . . . . . .  . . . . . . . . . .  . . . . . . . . . .  . . . . . . . . . .  . . .  259
```

Figure S3. Sequence alignments for *sed*.

Table S1. Primers used in this study.

Target	Primer ID	Primer Sequence (5′–3′)	Annealing Temperature (°C)
seb promoter	seb_p_fwd	TGAGGAGATTGAAGCAACGC	56
seb	seb_p_rev	AGGCAGGTACTCTATAAGTGCC	61
	seb_fwd	TGATGATAATCATGTATCAGCA	
	seb_rev	ACGGCGACACAGTAACTATCCA	
sec promoter	sec_p_fwd	TCAAGATGCTTAGAAATCCTCTGT	66
sec	sec_p_rev	AGCTTTCTGGAAGACCGTATCCTGT	63
	sec_fwd	TCAAGATGCTTAGAAATCCTCTGT	
	sec_rev	TCGGTGCTTGCCTTTTTAGGA	
sed promoter	sed_p_fwd	ATCCGGCTGTTCCTATGGCACC	56
sed	sed_p_rev	AGATGCACAAGCAAGGCGCT	62
	sed_fwd	TTCGAAATGCTGATGGTTGT	
	sed_rev	AGCTATCATCAATTTCTTTTCAAGC	
